# Control of motor output during steady submaximal contractions is modulated by contraction history

**DOI:** 10.1007/s00221-023-06774-8

**Published:** 2024-01-23

**Authors:** Abdulkerim Darendeli, Roger M. Enoka

**Affiliations:** 1 Movement Neuroscience Laboratory, Department of Physical Therapy, Movement, and Rehabilitation Sciences, Northeastern University, Boston, MA 02115 USA; 2 Faculty of Sport Sciences, Sivas Cumhuriyet University, Sivas, Turkey; 3 Department of Integrative Physiology, University of Colorado Boulder, Boulder, CO USA

**Keywords:** Force steadiness, Muscle contraction, EMG power density spectrum, Neural drive to muscle, Synaptic input

## Abstract

The purpose of the study was to investigate the influence of contraction history on force steadiness and the associated EMG activity during submaximal isometric contractions performed with the dorsiflexor muscles. The key feature of the protocol was a triangular ramp contraction performed in the middle of a steady contraction at a lower target force. The target force during the ramp contraction was 20% MVC greater than that during the steady contraction. Thirty-seven healthy individuals (21 men and 16 women) performed the submaximal tasks with the ankle dorsiflexors. Electromyography (EMG) signals were recorded from tibialis anterior with a pair of surface electrodes. The coefficient of variation for force was significantly greater during the second steady contraction compared with the first one at each of the seven target forces (*p* < 0.015; *d* = 0.38–0.92). Although the average applied force during the steady contractions before and after the triangular contraction was the same (*p* = 0.563), the mean EMG amplitude for the steady contractions performed after the triangular contraction was significantly greater at each of the seven target forces (*p* < 0.0001; *d* = 0.44–0.68). Also, there were significant differences in mean EMG frequency between the steady contractions performed before and after the triangular contraction (*p* < 0.01; *d* = 0.13–0.82), except at 10 and 20% MVC force. The greater force fluctuations during a steady submaximal contraction after an intervening triangular contraction indicate a change in the discharge characteristics of the involved motor units.

## Introduction

When performing a steady isometric contraction, the applied force fluctuates about an average value (Galganski et al. 1993; Laidlaw et al. 2000; Negro et al. 2016). The coefficient of variation for force—the normalized amplitude of these force fluctuations—provides a measure of force steadiness. Two features of the underlying motor unit activity can contribute to the amplitude of the force fluctuations: variability in the interspike intervals of individual motor units and the common low-frequency oscillations in discharge rate of the involved motor units (Moritz et al. 2005; Negro et al. 2009). Experimental measurements and computer simulations suggest that the fluctuations in force at low target forces is primarily attributable to the variability in interspike intervals, whereas the common low-frequency oscillations in discharge rate are responsible for the force fluctuations over most of the operating range of a muscle (Dideriksen et al. 2012; Farina and Negro 2015). However, experimental measures indicate marked overlap in the target forces at which the two measures of motor unit activity are significantly correlated with the fluctuations in force (Tsatsaki et al. 2022).

In addition to these discharge characteristics, force fluctuations can be modulated by the contraction history of the involved motor units. For example, evidence indicates that the discharge of action potentials by motor units can be maintained by persistent inward currents even after the termination of depolarizing synaptic input (Afsharipour et al. 2020; Gorassini et al. 1998; Heckman et al. 2008; Hounsgaard et al. 1984; Lee and Heckman 1998). The influence of this intrinsic motor neuron property on motor unit activity was demonstrated by Beauchamp et al. (2023) when they compared the discharge of motor units in the tibialis anterior and medial gastrocnemius muscles during steady contractions at 10% MVC force that were performed before and after an intervening triangular contraction to a greater target force. Many of the motor units recruited during the triangular contraction sustained discharge when performing the second steady contraction, which reduced the average discharge rate of the entire pool. These adjustments are expected to influence the coefficient of variation for force during the steady contractions, which is what we examined in our study.

The purpose of our study was to investigate the influence of contraction history on force steadiness and the associated EMG activity during submaximal isometric contractions performed with the dorsiflexor muscles. We used the protocol developed by Beauchamp et al. (2023) to compare the coefficient of variation for force and a global measure of muscle activation (mean amplitude and frequency of the surface EMG) during the steady contractions performed before and after a triangular contraction. As an extension of this protocol, we compared the effects for seven target forces performed with the dorsiflexor muscles. We hypothesized that the coefficient of variation for force (force steadiness) during the submaximal isometric contractions would increase after an intervening triangular ramp contraction.

## Methods

### Participants

This study was approved by the Institutional ethics committee (reference no. 2023-06/02) and conducted in accordance with the Declaration of Helsinki. Thirty-seven healthy individuals (21 men and 16 women; 22.8 ± 5.5 years, height 1.7 ± 0.1 m, body mass 70 ± 16 kg) were recruited for the study. Participants self-reported no history of neurological or musculoskeletal disorders. Possible benefits and risks of the study were explained prior to providing written informed consent. Participants were required to abstain from alcohol, caffeine, and high-intensity exercise for 24-h before their visit.

### Experimental procedures

**Fig. 1:**
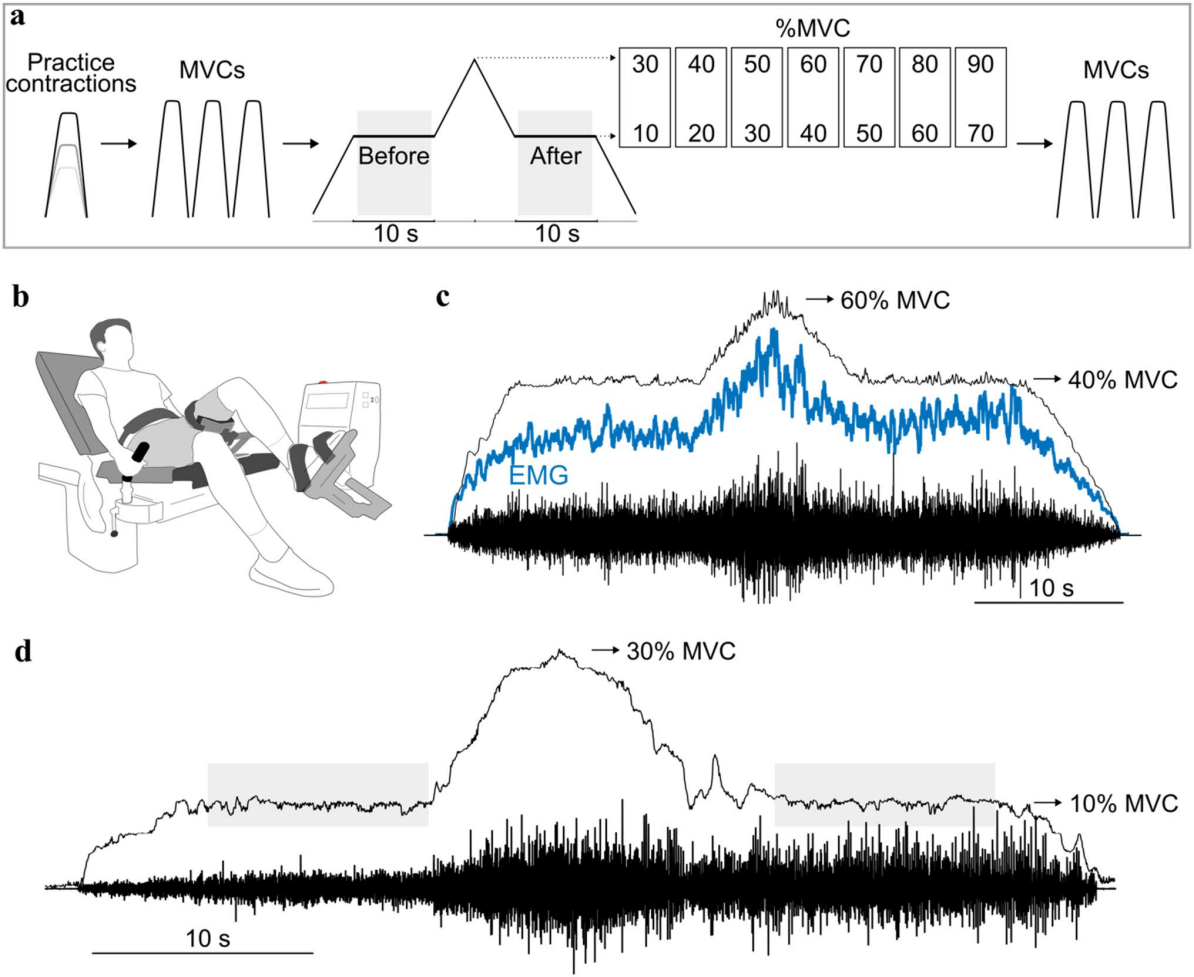
Experimental protocol. (**a**) The order of the measurements during the single visit. Maximal voluntary contractions (MVC) with the dorsiflexors were performed before and after the submaximal tasks. The primary task was a sombrero contraction, which involved a triangular ramp contraction performed in the middle of a steady contraction. The steady contractions were performed in a counterbalanced order at 7 target forces: 10, 20, 30, 40, 50, 60, and 70%. The target force during the ramp contraction was 20% greater than that for the steady contraction. The steady contractions each lasted ~10 s. (**b**) Participant position for ankle dorsiflexion tasks. (**c**) An example of the low-pass filtered force (*black*), smoothed EMG (*blue*), and interference EMG data collected for one participant. The steady contraction was performed at 40% MVC and the peak triangular force was 60% MVC. (**d**) As in **c** but for a different trial in which the two target forces were 10 and 30% MVC. *Gray-shaded* regions along the horizontal axis denote the ~10 s windows used for data analysis (Colour figure online)

Participants were informed about the experimental protocol upon arrival at the laboratory. After being secured in an isokinetic dynamometer (IsoMed 2000, D&R, Ferstl, Hemau, Germany), participants practiced the experimental task. This involved a triangular ramp up-and-down contraction performed in the middle of several submaximal isometric contractions with the ankle dorsiflexors. Maximal voluntary contractions (MVC) were performed before and after the submaximal tasks (Fig. 1). EMG electrodes were attached to the skin over the tibialis anterior muscle.

The submaximal isometric contraction comprised a 5 s ramp up to the target force, a 10 s steady contraction, a 5 s ramp up to a target force that was 20% MVC force greater than the preceding steady contraction, a 5 s ramp back down to the lower target force, another 10 s steady contraction, and 5 s ramp down to baseline. The force trajectory during this task is known as a sombrero contraction (Beauchamp et al. 2023). The primary outcome variable was the coefficient of variation for force (force steadiness) during the steady contractions before and after the triangular contraction. When performing the tasks, visual feedback was provided on a computer screen placed in front of the participant. The vertical axis of the screen was proportional to the force registered by the isokinetic dynamometer. Participants were instructed to reach the target force displayed on the screen and maintain a steady contraction.

The isometric contractions were performed with the dorsiflexors at an ankle and knee angles of 90° (Fig. 1b). The seven target forces (Fig. 1a) for the steady contractions were set relative to the MVC force: 10, 20, 30, 40, 50, 60, and 70% MVC force. The steady contractions at the different target forces were performed in a counterbalanced order across participants, with 120 s of rest between each trial.

Two sets of MVCs (three trials each) for the ankle dorsiflexors were performed before and after the steady contractions. Each MVC trial lasted ~6 s and involved a 3 s increase from rest up to maximum that was held for 3 s. There was 2 min of rest between trials. Strong verbal encouragement was provided during each MVC trial.

### EMG recordings

A wireless surface EMG system (Ultium EMG, Noraxon USA Inc., Scottsdale, AZ) was used to record muscle activity of tibialis anterior. The signals were sampled at 2000 Hz and band-pass filtered (10–500 Hz). The skin over tibialis anterior was shaved and cleaned with abrasive gel (Nuprep; Weaver and Company, Aurora, CO) and water. A pair of disposable Ag/AgCl self-adhesive gel electrodes (diameter of 1.3 cm, Noraxon Product No. 272; Noraxon USA Inc.) were attached to the skin with 2 cm between the centers of the two electrodes. The EMG and force signals were synchronized using an analog input system (16 channel BNC, Noraxon USA Inc. Scottsdale, AZ) and transferred to the same computer.

### Data analysis

All data were processed in myoMuscle software (Noraxon USA Inc. Scottsdale, AZ) and MATLAB (2021b, MathWorks, Natick, MA). Mean EMG amplitude and frequency along with the coefficient of variation for force were calculated for the steady contractions performed before and after each triangular ramp contraction. The force signal was low-pass filtered (20 Hz, fourth-order, Butterworth). Subsequently, the coefficient of variation for force was calculated (standard deviation/mean × 100) for each 10 s steady contraction.

The root mean square (RMS, with 250-ms moving windows) value of the high-pass filtered (20 Hz, fourth-order, Butterworth) EMG signal was calculated. Mean EMG amplitude was quantified as the average of RMS value during each 10 s steady contraction and normalized to the maximal EMG amplitude during the initial MVC. The mean EMG frequency for the submaximal steady contractions was calculated across 1-s non-overlapping windows. Mean frequency was quantified as the frequency that corresponded to the peak value in the power density spectrum derived from the interference EMG signal.

### Statistics

All statistical analyses were performed in R (version 4.3.1; R core team, Vienna, Austria) using rstatix library (version 0.7.2). The normality of the distributions and the equality of variances for the outcome variables were confirmed using the Shapiro–Wilk test and the Levene’s test, respectively. A two-way repeated-measures ANOVA was used to evaluate the differences in the coefficient of variation for force and the EMG measures across the target forces (10–70% MVC force) and the 10 s steady contractions (before and after the triangular contraction). Holm’s correction was applied to adjust *p* values for pairwise comparisons. MVCs performed before and after the submaximal tasks were compared using paired *t *tests.

Effect sizes (Cohen’s d) were calculated to indicate the magnitude of the difference in outcome variables between the steady contractions performed before and after the triangular contraction at each target force. The effect size thresholds were as follows: |*d*| < 0.2 negligible, |*d*| < 0.5 small, |*d*| < 0.8 medium and |*d*| ≥ 0.8 large (Cohen 1992).

## Results

It seems unlikely that the protocol reduced the force capacity of the dorsiflexor muscles as each trial lasted approximately 40 s and was followed by a rest period of 120 s. Consistent with this expectation, MVC torque for the dorsiflexors after all the submaximal contractions (26.6 ± 8.0 Nm) was not statistically different (*p* = 0.46) from that at the start of the protocol (26.5 ± 8.0 Nm).

Examples of the force and EMG signals recorded during this experiment are plotted in Fig. 1. One notable feature of our observations was that many participants found it challenging to reduce the applied force back to the lower target force after the triangular contraction, especially at lower target forces. For example, the applied force shown in Fig. 1d fluctuated above the target force for approximately 3 s before settling on the target. We analyzed only the steady portions of the contractions at each target force (Fig. 1d, gray-shaded regions).

### Decrease in force steadiness

**Fig. 2:**
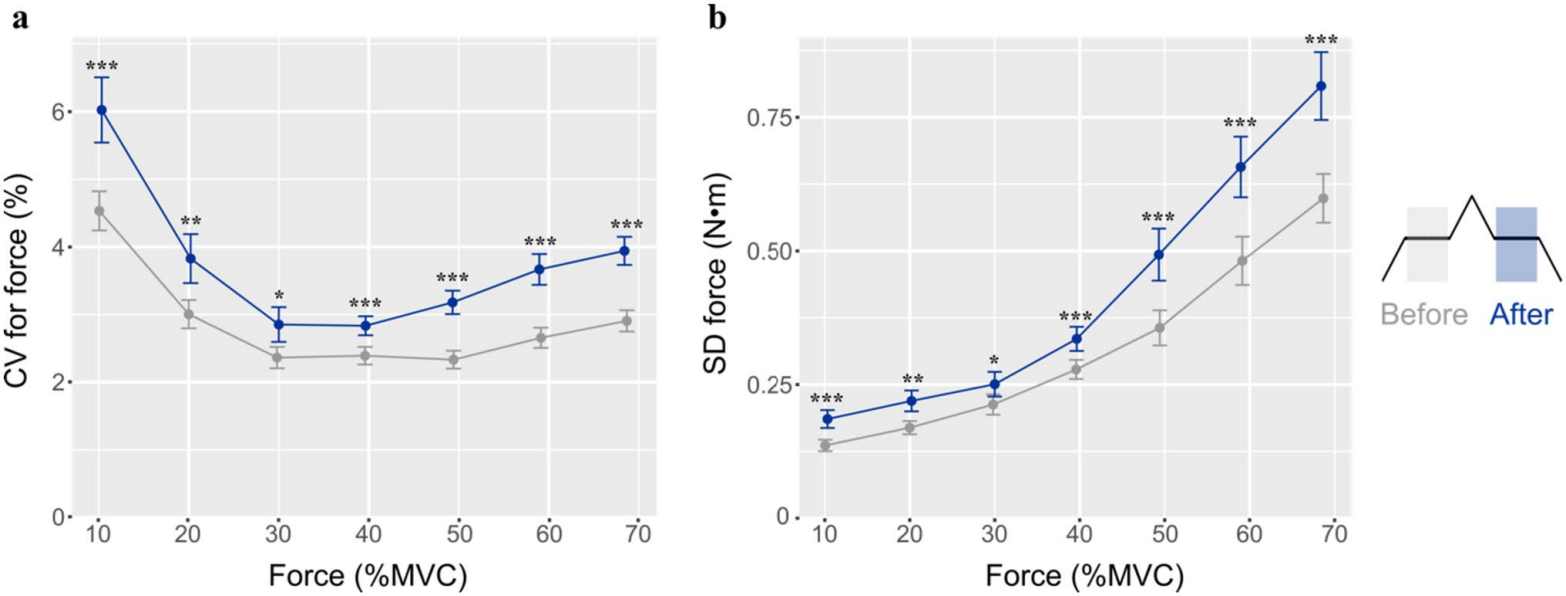
Amplitude of force fluctuations quantified as (**a**) coefficient of variation (CV) for force and (**b**) standard deviation (SD) for force during the 10 s submaximal steady contractions at the 7 target forces before (*gray*) and after (*blue*) the triangular ramp contraction. Data are mean ± standard error. ^*^p < 0.05; ^**^p < 0.01; ^***^p < 0.001 compared with the Before values (Colour figure online)

The target force for the steady contractions performed before and after the triangular contraction was not statistically different (*p* = 0.563). However, the coefficient of variation for force was significantly greater during the second steady contraction compared with the preceding one at each of the 7 target forces (Fig. 2). The effect size for the difference in the coefficient of variation for force between the two steady contractions was greatest at the lowest (10%: *d* = 0.62) and three highest (50%: *d* = 0.90; 60%: *d* = 0.86; and 70%: *d* = 0.92) target forces (Table 1).

**Table 1:** Mean and 95% confidence intervals (CI) for coefficient of variation for force during the 10 s steady contractions performed at seven target forces before and after the triangular ramp contraction

	Target force						
	10%	20%	30%	40%	50%	60%	70%
Before (95% CI)	4.5 (3.9–5.1)	3 (2.6–3.4)	2.4 (2–2.7)	2.4 (2.1–2.7)	2.3 (2.1–2.6)	2.7 (2.4–3)	2.9 (2.6–3.2)
After (95% CI)	6.0 (5.0–7.0)	3.8 (3.1–4.6)	2.9 (2.3–3.4)	2.8 (2.5–3.1)	3.2 (2.8–3.5)	3.7 (3.2–4.1)	3.9 (3.5–4.4)
*p* ∣*d*∣	0.0004 0.62	0.005 0.46	0.015 0.38	0.0003 0.54	0.0000002 0.90	0.0000009 0.86	0.000005 0.92

The main effect of contraction intensity on the coefficient of variation for force was also statistically significant. The coefficient of variation for force during the initial steady contraction (before) was significantly different for two comparisons: (1) 10% MVC *vs.* the other six target forces (*p* < 0.001), and (2) between 30 and 70% MVC (*p* = 0.025). Similarly, the coefficient of variation for force during the second steady contraction (after) was significantly greater at 10% MVC than at the other six target forces (*p* < 0.01). The other statistically significant differences in the coefficient of variation for force for the steady contractions performed after the triangular contraction included: (1) 70 vs. 50% (*p* = 0.019), 40% (*p* < 0.0001), and 30% MVC (*p* = 0.003); (2) 60 vs. 40% (*p* = 0.02) and 30% MVC (*p* = 0.032); and (3) 20 vs. 30% MVC (*p* = 0.03).

### Increase in EMG amplitude

**Fig. 3:**
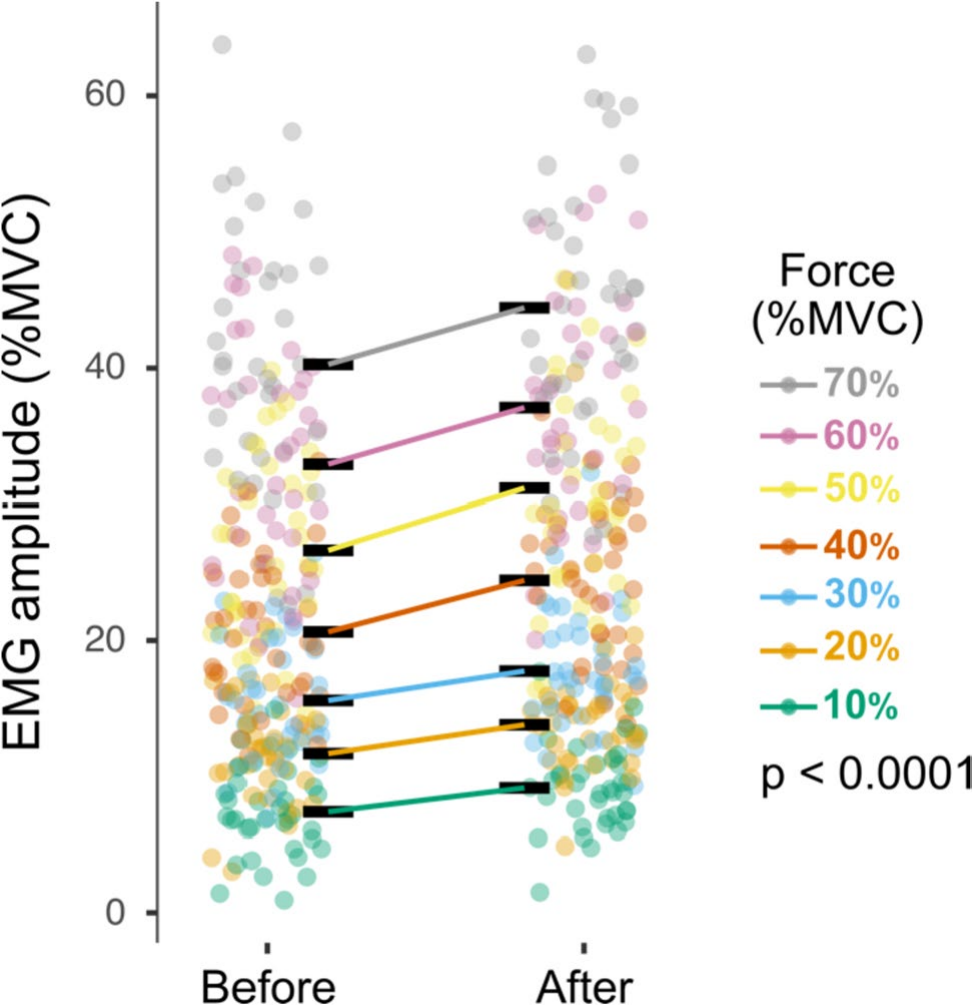
Mean EMG amplitude (%MVC) for tibialis anterior during the 10 s steady contractions performed before and after the triangular ramp contraction. *Each dot* indicates the EMG value for one participant with each target force (%MVC) indicated in a different color. *Black lines* show the average EMG amplitude at each target force. The difference in EMG amplitude before and after the ramp was significantly different at each target force (*p* < 0.0001)

Mean EMG amplitude during the 10-s steady contractions performed after the triangular contraction was significantly greater than that during the initial steady contraction at each of the 7 target forces (*p* < 0.0001) (Fig. 3). The increase in EMG amplitude averaged 3.24 ± 1.2% MVC [95% CI: (2.16 4.33)] across the seven target forces. The elevated EMG amplitude, however, did not influence the applied force as this did not differ between the pairs of steady contractions (*p* = 0.563). The effect sizes for the differences in mean EMG amplitude between the pairs of steady contractions was moderate at 10% (*d* = 0.56), 20% (*d* = 0.6), 40% (*d* = 0.68), and 50% MVC (*d* = 0.63), whereas it was less at 30% (*d* = 0.48), 60% (*d* = 0.49), and 70% MVC (*d* = 0.44).

### Decrease in mean EMG frequency

**Fig. 4:**
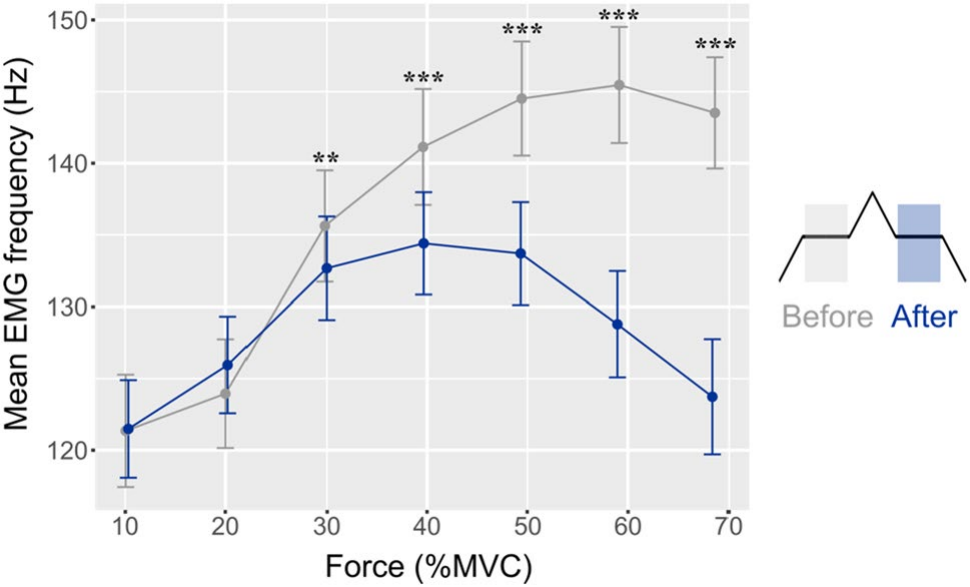
Mean EMG frequency (Hz) during the 10 s steady contractions at the 7 target forces before (*gray*) and after (*blue*) the triangular ramp contraction. Data are mean ± standard error. ^**^p < 0.01; ^***^p < 0.001 compared with the Before values (Colour figure online)

Mean EMG frequency during the first steady contraction increased progressively with target force up to the second highest target force (60%). After the triangular contraction, however, the peak mean EMG frequency occurred at the 40% target force and the value for all subsequent target forces was less than that for the first steady contraction (Fig. 4). The difference in mean EMG frequency between the pairs of steady contractions was statistically significant at the target forces of 30% (*p* < 0.01; *d* = 0.13), 40, 50, 60, and 70% MVC (*p* < 0.001; *d* = 0.29–0.82). Also, the magnitude of the difference in mean EMG frequency between the two steady contractions increased with force level; for example, the difference was ~7 Hz at 40% MVC force (*d* = 0.29), whereas it was ~20 Hz at 70% MVC force (*d* = 0.82).

## Discussion

The main finding of the study was that the coefficient of variation for force, EMG amplitude for tibialis anterior, and the mean frequency of the interference EMG signal during the steady submaximal contraction were all altered by an intervening triangular contraction. Despite the mean applied force not differing during the first and second steady contractions, both the coefficient of variation for force and mean EMG amplitude were greater during the second steady contraction. Also, the mean EMG frequency did not increase progressively across target forces during the second steady contraction, as it did during the steady contractions performed before the triangular contraction.

### Force steadiness

Although contraction history can influence muscle force and that of its constituent motor units (Burke et al. 1976; Gorassini et al. 1998; Proske et al. 2014; Rassier and Herzog 2004; Shi et al. 2023; Van Cutsem and Duchateau 2005), its impact on the normalized force fluctuations (force steadiness) largely reflects changes in the modulation of discharge rate (Dideriksen et al. 2012; Martinez-Valdes et al. 2022; Mazzo et al. 2022; Nagamori et al. 2021; Thompson et al. 2018). Our finding of an increase in the coefficient of variation for force during the second steady contraction replicates the result reported by Beauchamp et al. (2023). In their study, the sombrero contraction went from a steady contraction of 10% MVC torque to a peak triangular torque of 30% MVC, and the task was performed with both the plantar flexor and dorsiflexor muscles. In both studies, the average force was not statistically different between the first and second steady contractions. Our results extend those of Beauchamp et al. by demonstrating the decline in steadiness (coefficient of variation for force) was evident at all the target forces we examined, with the greatest effect at the lowest (10%) and four highest (40–70%) target forces.

Previous studies suggest that the greater fluctuations in force during the second steady contraction were attributable to either an increase in the coefficient of variation for interspike interval or an increase in the amplitude of the common low-frequency oscillations in discharge rate (Dideriksen et al. 2012; Farina and Negro 2015; Moritz et al. 2005; Negro et al. 2009). The significant adjustments in motor unit activity during the steady contraction performed after the triangular contraction are presumably responsible for the increase in the coefficient of variation for force (Beauchamp et al. 2023). This association needs to be examined explicitly. Moreover, it would be of interest to determine if the adjustments in discharge activity are accompanied by changes in the distribution of common synaptic input to the motor neuron pool as assessed by the identification of motor unit modes (Del Vecchio et al. 2023; Levine et al. 2023; Ricotta et al. 2023).

### EMG amplitude

The second main finding was the greater EMG amplitude during the second steady contraction relative to the one performed before the triangular ramp contraction. The elevated EMG amplitude can be explained by the results of Beauchamp et al. (2023) who decomposed high-density EMG recordings into the discharge times of action potentials by many concurrently active motor units. They identified three groups of motor units: one group was activated during the first steady contraction, a second group was only active during the triangular ramp contraction, and a third group was recruited during the triangular contraction and remained active during the second steady contraction. This activity pattern was observed for motor units in tibialis anterior during dorsiflexion and in medial gastrocnemius during plantar flexion. The sustained discharge of the third group of motor units was attributed to the activation of persistent inward currents and was presumably responsible for the increase in EMG amplitude in our study.

Although statistically significant, the increase in EMG only averaged 3.24 ± 1.2% MVC across the seven target forces. This adjustment did not influence the average force during the second steady contraction, which did not differ from that during the first steady contraction. Nonetheless, the sustained discharge of the third group of motor units observed by Beauchamp et al. must have contributed to the increase in the coefficient of variation for force. Our results did not provide insight on this issue, but previous studies suggest that the greater fluctuations in force during the second steady contraction were attributable to either an increase in the coefficient of variation for interspike interval or an increase in the amplitude of the common low-frequency oscillations in discharge rate (Dideriksen et al. 2012; Farina and Negro 2015; Moritz et al. 2005; Negro et al. 2009).

As the final common pathway for the transformation of an activation signal into contractile force, motor unit activity establishes the quantity and quality of the resulting muscle force. Beauchamp et al. (2023) found that the target force during the second steady contraction was achieved with a reduction in the average discharge rate in combination with motor units that were recruited during the triangular contraction. Average discharge rate for tibialis anterior motor units was approximately 10 pps during the first steady contraction and this declined by 1.75 pps during the second plateau, both of which are rather low on the sigmoidal relation between discharge rate and force (Macefield et al. 1996). This decline in discharge rate was accompanied by the recruitment of an average of five motor units during the second steady contraction. Nonetheless, these adjustments were sufficient to achieve the target torque. What remains unknown, however, is those discharge characteristics that were responsible for the increase in the coefficient of variation for force.

### Mean EMG frequency

The third main finding was a shift in the mean frequency for the interference EMG signal during the second steady contraction. Interpretation of changes in the power density spectrum is challenging (Farina et al. 2004). Although an interference EMG signal comprises the sum of muscle fiber action potentials (Dideriksen et al. 2011), its mean amplitude is relatively insensitive to modest changes in motor unit activity (Christie et al. 2009; Farina et al. 2014). Moreover, the spectral content of the interference EMG signal cannot detect either motor unit recruitment or average discharge rate (Del Vecchio et al. 2017; Dideriksen and Farina 2019; Farina et al. 2004), and the mean frequency is not associated with the amount of motor unit activity (Farina et al. 2014). Rather, the mean frequency is most influenced by the shape of the intracellular action potential and the impact of the volume conductor on the shape of motor unit action potentials (Boonstra and Breakspear 2012; Dideriksen et al. 2011; Farina et al. 2014).

Given the minimal demands of our protocol, the change we observed in mean frequency across the seven target forces likely reflect changes in the average shape of motor unit action potentials. Accordingly, the increase in mean frequency across target forces during the first steady contraction suggest a compression in the time domain of the average shape of the motor unit action potentials, presumably due to the recruitment of high-threshold motor units (Del Vecchio et al. 2017; Keenan et al. 2005; Milner-Brown and Stein 1975). In contrast, the profile differed during the second steady contraction with the mean frequency declining at the two highest target forces (60 and 70% MVC) despite a progressive increase in EMG amplitude. Potential explanations for this finding include the recruitment of motor units further away from the recording electrode or a change in the proportion of motor units that were recruited during the triangular contraction and sustained discharge into the second steady contraction. This issue needs to be resolved by recording the activity of motor units with grid electrodes during strong sombrero contractions.

### Future directions

Our study has only explored one part of the contraction-history landscape and our results are not intended to generalize across conditions. For example, subsequent studies should examine the influence of joint angle, manipulate the features of the intervening triangular contraction, identify the motor unit discharge characteristics that are responsible for the reduction in force steadiness, and evaluate the functional consequences of the increase in force fluctuations during submaximal isometric contractions. This protocol, which was developed by Beauchamp et al. (2023), provides an opportunity to increase our knowledge of motor unit physiology.

In conclusion, control of force applied by the dorsiflexor muscles during a steady submaximal contraction was compromised by a prior ramp-up and ramp-down (triangular) isometric contraction. Although participants were able to match the seven target forces before and after the triangular contractions, the coefficient of variation for force and EMG amplitude for tibialis anterior were greater during the second steady contraction. Moreover, the mean frequency for the interference EMG signal suggested that there was a change in the involved motor units during the second steady contraction. This work, however, underscores the need for more studies on the influence of contraction history on motor unit physiology.

## Data Availability

Data will be made available by the authors upon reasonable request.
